# Genetic evidence of Dobrava virus in Apodemus agrarius in Hungary.

**DOI:** 10.3201/eid0503.990324

**Published:** 1999

**Authors:** J J Scharninghausen, H Meyer, M Pfeffer, D S Davis, R L Honeycutt

**Affiliations:** Texas A&M University, College Station 77843, USA. 100304.2713@compuserve.com

## Abstract

Using nested polymerase chain reaction, we sequenced Dobrava virus (DOB) from the rodent Apodemus agrarius in Hungary. The samples we isolated group with DOB samples previously isolated from A. flavicollis. This grouping may indicate host switching.


### Genetic Evidence of Dobrava Virus in *Apodemus agrarius* in Hungary

**Jerrold J. Scharninghausen,* Hermann Meyer,† Martin Pfeffer,‡ Donald S. Davis,* and Rodney L. Honeycutt***
*Texas A&M University, College Station, Texas, USA;
†Federal Armed Forces Medical Academy, Munich, Germany; and
‡Ludwig Maximilians University, Munich, Germany

Using nested polymerase chain reaction, we sequenced Dobrava virus (DOB) from the rodent *Apodemus agrarius* in Hungary. The samples we isolated group with DOB samples previously isolated from *A. flavicollis*. This grouping may indicate host switching.

Hantaviruses, the causative agents of hemorrhagic fever with renal syndrome and hantavirus pulmonary syndrome, are serologically related viruses of the family *Bunyaviridae* and have a worldwide distribution. Unlike other bunyaviruses, hantaviruses are not transmitted by arthropod vectors. The virus is excreted in the saliva, urine, and feces of infected rodents. Humans become infected by inhalation of aerosols of dried excreta, inoculation through the conjunctiva, or entry through broken skin (1).

Each viral species within the genus *Hantavirus* is primarily associated with a single rodent species, although accidental infections have been reported in other mammals (2). Four primary reservoirs for hantaviruses are found in Europe: *Rattus norvegicus,* Seoul virus; *Clethrionomys glareolus,* Puumala virus (PUU); *Apodemus flavicollis,* Dobrava virus (DOB); and *Microtus arvalis,* Tula virus (TUL) (3). An additional rodent species, *Apodemus agrarius,* is the primary reservoir of Hantaan virus (HTN), the causative agent of Korean hemorrhagic fever, which is found throughout Korea and China but not in Europe. Recently, DOB was isolated from *A. agrarius* in Estonia (4) and Russia (5).

Hantaviruses are present in Hungary; however, their particular virus strains or species and their distribution are unknown. PUU has been confirmed in western Hungary, but the species in eastern Hungary have not been determined (6).

Address for correspondence: Jerrold J. Scharninghausen, Department of Wildlife & Fisheries Sciences, Texas A&M University, College Station, Texas 77843, USA; e-mail: 100304.2713@compuserve.com.

During field surveys of indigenous rodents in areas used by NATO forces between 1995 and 1996, nine rodents were collected at Tazar Air Force Base, outside Kaposvar, eastern Hungary. A total of 210 trap nights resulted in a trap return of 4.28%. Two *A. agrarius*, four *Microtus agrestis*, and three *Mus musculus* were captured. Animals were live-trapped and euthanized with halazone. Tissue was collected from lungs and kidneys; urine, if present, was also collected.

Total RNA was extracted from collected tissues by using the FastRNA Kit–Green (Bio 101), according to the supplier's recommendations. RNA was converted to cDNA and amplified by the Titan One Tube RT-PCR System (Boehringer, Mannheim, Germany), according to the manufacturer's recommendations. Degenerate primers M-4 (ATGAARGCNGAWGA RNTNACMCCNGG) and M-9 (TGRYCNAGYTG TATYCCCATWGATTG) were used to amplify a 583-bp section corresponding to nt positions 605 to 1,188 in the S segment of HTN strain X95077. No DOB strains were used in the laboratory.

A target sequence of 397 bp (nt from 692 to 1,089) was amplified by nested polymerase chain reaction, and both strands of the amplicons were sequenced. Amplified bands of the predicted size were demonstrable in all tissue samples investigated from the two specimens of *A. agrarius* (designated Tazar-2, Tazar-8). All other samples tested negative. Sequence validity was confirmed by sequencing amplified lung and kidney products from each infected animal. The respective sequences from Tazar-2 and Tazar-8 differed in 3 of the 321 nucleotides examined

(99% identity). Both sequences were aligned with the corresponding S-segment section of several DOBs and five other European and Asian hantaviruses.

The nucleotide sequence identities between Tazar virus (found in this study) and related viral lineages (Figure) included Russian DOB from *A. agrarius,* 88%; Estonian DOB from *A. agrarius,* 86%-87%; Bosnia DOB from *A. flavicollis,* 88%; Greek DOB from *A. flavicollis,* 88%-85%; Sapporo rat virus, 70%; HTN, 68%; Khabarousk, 57%; PUU, 56%; TUL, 53%; and Sin Nombre, 48%. These results indicate that, on the basis of sequence similarity, both Tazar samples are hantaviruses most closely related to DOB. Although the sequence data are limited, phylogenetic analyses linked Tazar-2 and Tazar-8 isolated from *A. agrarius* to a group of DOB previously isolated from *A. flavicollis.* The *A. agrarius* DOB from Russia (5) did not support monophyly (common ancestry) for DOB isolated from *A. agrarius* populations in Hungary and Russia. Other representative hantaviruses, including PUU, TUL, HTN, and Sapporo rat virus, were more basal in the phylogeny (Figure).

HTN infects *A. agrarius* populations in Asia but has not been isolated in Europe. Direct enzyme-linked immunosorbent assay has demonstrated the presence of Hantaan-like antigens in *A. agrarius* in the former republic of Czechoslovakia (5.5%, [7]), the European regions of the former Soviet Union (5.3%, [8]) (28.5%, [9]), and Serbia (2.2%, [10]). Because HTN sequences have not been reported in Europe and HTN and DOB have similar immunologic responses, the earlier findings of Hantaan-like antigens in Europe may represent a more widespread occurrence of DOB in European populations of *A. agrarius*. Recent verification of DOB in populations of *A. agrarius* in Estonia (4), Russia (5), and now Hungary supports this conclusion.

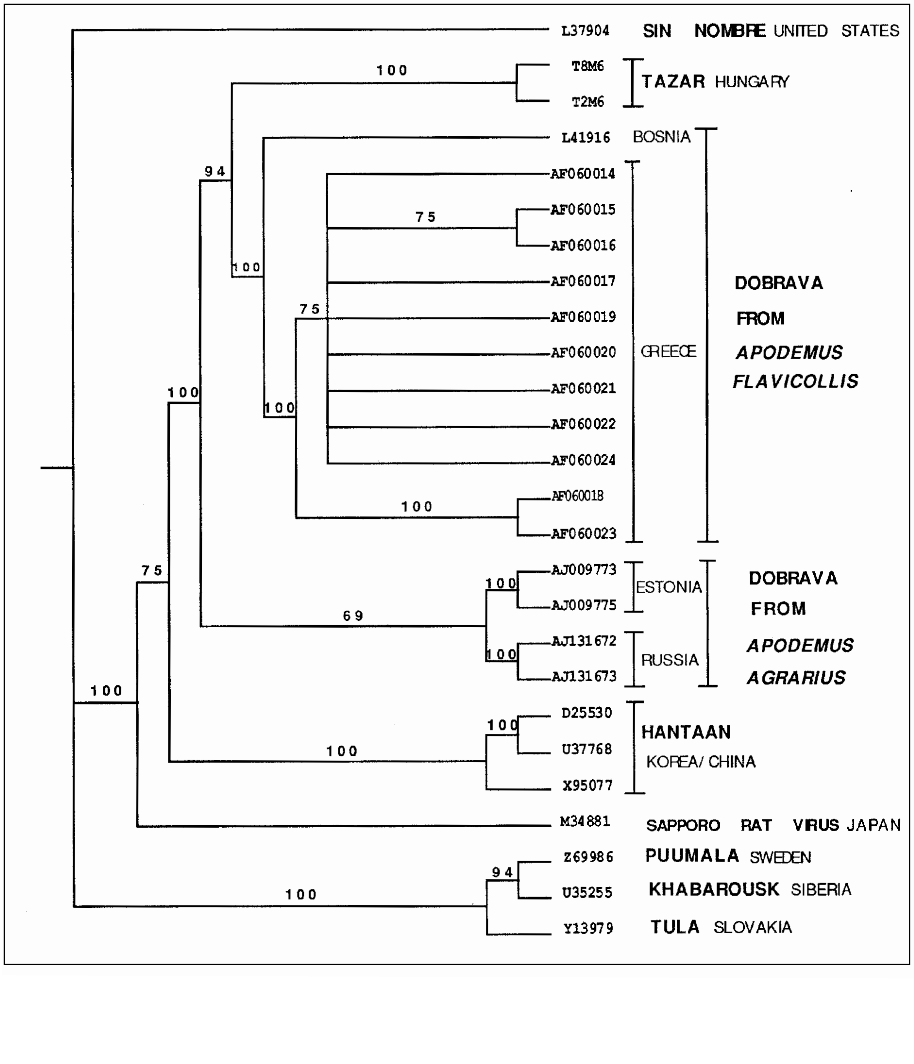

Figure. Cladogram derived from nucleotide sequences of Tazar-2, Tazar–8, and other hantaviruses. Numbers denote Genbank accession numbers. The cladogram was derived from the neighbor-joining estimated phylogeny and bootstrap analysis using p-distance estimates. The phylogenetic analysis was performed by using PAUP 3.1.1 (vers. 4.0.0d64). Numbers at each internode or bifurcation represent bootstrap support based on 1,000 replicates. A Sin Nombre virus sequence (L37904) was used as the outgroup to root the tree. Tazar 2 and Tazar 8 have been submitted to Genbank and received accession numbers AF085336 and AF085337, respectively.

The occurrence of DOB in both *A. agrarius* and *A. flavicollis* provides an opportunity to evaluate the hypothesis concerning distribution of hantaviruses in related rodent hosts. Phylogenetically different Sin Nombre–like viruses have been found in different populations within species of peromyscine rodents that vary ecologically and geographically throughout their range (11). Although these authors found evidence of cospeciation between the rodent host phylogeny and the host-borne hantavirus phylogeny, evidence of host switching was observed with *Peromyscus leucopus*–borne New York virus grouped with *P. maniculatus*–borne viruses rather than with other *P. leucopus*–borne viruses.

Our phylogenetic analysis (Figure) indicates a closer relationship between the *A. agrarius* DOB from Hungary and *A. flavicollis* DOB, with Russian/Estonian DOB representing a sister-group to this clade. This lack of monophyly for the *A. agrarius* DOB may suggest host switching between *A. agrarius* and *A. flavicollis* similar to that between *P. leucopus* and *P. maniculatus*. Nevertheless, the relationships between the isolated DOB lineages suggest a more basal position for the *A. agrarius* DOB than for *A. flavicollis* DOB lineages.

*A. flavicollis* ranges throughout much of Western Europe eastward to the Ural Mountains, and *A. agrarius* ranges from Eastern Europe eastward to the Pacific Ocean, covering most of the Asian continent (12). Given the extensive range of both species, an examination of other populations within each species, as well as other species of *Apodemus*, might allow correlation of the viral and rodent host phylogeny. The pattern of divergence for hantaviruses in New World peromyscine rodent species may be mirrored in Old World arvicoline rodents. Therefore, the existence of more than one hantavirus in *A. agrarius* may reflect geographic variation within the species or host switching in regions where two host species are potentially sympatric.

Dr. Scharinghausen, an active-duty Army captain, is completing his Ph.D. at Texas A&M University in Wildlife and Fisheries Sciences. His area of expertise is mammalogy, and his research interests include zoonoses and molecular biology.

### References

1. Hjelle B, Jenison S, Goade D, Green W, Feddersen R, Scott A. Hantaviruses: clinical, microbiologic, and epidemiologic aspects. Crit Rev Clin Lab Sci 1995;32:469-508.
2. Childs J, Glass G, Korch G, LeDuc J. Prospective seroepidemiology of hantaviruses and population dynamics of small mammal communities of Baltimore, Maryland. Am J Trop Med Hyg 1987;37:648-62.
3. Plyusnin A, Cheng Y, Vapalahti O, Pejcoch M, Unar J, Jelinkova Z, et al. Genetic variation in Tula hantaviruses: sequence analysis of the S and M segments of strains from Central Europe. Virus Res 1995;39:237-50.
4. Nemirov K, Vapalahti O, Lundkvist A, Vasilen Golovljova I, Plyusnina A, Niemmimaa J, et al. Isolation and characterization of Dobrava hantavirus in the striped field mouse (*Apodemus agrarius*) in Estonia. J Gen Virol 1999;80:371-9.
5. Plyusnin A, Nemirov K, Apekina N, Plyusnina A, Lundkvist A, Vaheri A. Dobrava hantavirus in Russia. Lancet 1999;353:207.
6. Centers for Disease Control and Prevention. The Fourth International Conference on HFRS and Hantaviruses; 1998 March 5-7; Atlanta, Georgia. Atlanta: U.S. Department of Health and Human Services; 1998.
7. Danes L, Tkachenko E, Ivanov A, Lim D, Rezapkin G, Dzagurova T. Hemorrhagic fever with renal syndrome in Czechoslovakia: detection of antigen in small terrestrial mammals and specific serum antibodies in man. Journal of Hygiene, Epidemiology, Microbiology, and Immunology 1986;30:79-85.
8. Tkachenko E, Ivanov A, Donets M, Miasnikov Y, Ryltseva E, Gaponova L, et al. Potential reservoir and vectors of haemorrhagic fever with renal syndrome (HFRS) in the USSR. Annales de Société Belgique Medecíne Tropique 1983;63:267-9.
9. Gavrilovskaya I, Apekina N, Myasnikov Y, Brenshtein A, Ryltseva E, Gorbachkova E, et al. Features of circulation of hemorrhagic fever with renal syndrome (HFRS) virus among small mammals in the European USSR. Arch Virol 1983;75:313-6.
10. Gligic A, Obradovic M, Stojanovic R, Hlaca D, Antonijevic B, Arnautovic A, et al. Hemorrhagic fever with renal syndrome in Yugoslavia: detection of hantaviral antigen and antibodies in wild rodents and serological diagnosis of human disease. Scand J Infect Dis 1988;20:261-6.
11. Morzunov S, Rowe J, Ksiazek G, Peters CJ, St Jeor S, Nichol S. Genetic analysis of the diversity and origin of hantavirus in *Peromyscus leucopus* mice in North America. J Virol 1998;1:57-64.
12. Nowak RM, editor. Walker's mammals of the world. 5th ed. Baltimore: Johns Hopkins University Press; 1991.

